# Age-Related Increase in Type C Tympanogram

**DOI:** 10.3390/jcm13216324

**Published:** 2024-10-23

**Authors:** Taeuk Cheon, Ju Ha Park, Ji Seop Lee, Seong Hoon Bae

**Affiliations:** Department of Otorhinolaryngology, Gangnam Severance Hospital, Yonsei University College of Medicine, Seoul 06273, Republic of Korea; entcheon@yuhs.ac (T.C.); jhpark0727@yuhs.ac (J.H.P.); tjqto7@yuhs.ac (J.S.L.)

**Keywords:** aging, tympanometry, type C tympanogram, middle ear dysfunction, KNHANES

## Abstract

**Background:** This study evaluated the relationship between aging and tympanometric changes, specifically the incidence of type C tympanograms, using data from the Korea National Health and Nutrition Examination Survey (KNHANES). **Methods:** We analyzed data from the 2019–2022 KNHANES, including 26,738 ears from individuals aged 40 and older. Tympanometry at 226 Hz identified type C tympanograms based on peak pressure below −100 daPa. Multiple logistic regression evaluated the association between age and type C tympanograms, adjusting for confounders such as sex, smoking status, allergic rhinitis, chronic sinusitis, and lifestyle disease markers. **Results:** The incidence of type C tympanograms increased significantly with age (R^2^ = 0.953, *p* < 0.001). Age was the most significant factor associated with type C tympanograms (*p* < 0.001, odds ratio 1.052), while other factors were not statistically significant. Ears with type C tympanograms had significantly poorer hearing thresholds compared to those with type A tympanograms (*p* < 0.001). **Conclusions:** Aging is significantly associated with an increased incidence of type C tympanograms, indicating possible middle ear dysfunction in older adults. The correlation between type C tympanograms and poorer hearing thresholds suggests that age-related changes in middle ear function may contribute to hearing impairment. Tympanometric screening in older adults may facilitate early detection and management of middle ear dysfunction, potentially improving hearing outcomes and quality of life.

## 1. Introduction

Age-related changes in the human body, particularly those affecting the nervous system, often result in functional decline. Age-related hearing loss (ARHL), also known as presbycusis, is one of the most well-documented examples of this phenomenon and is characterized by progressive sensorineural hearing impairments [1,2,3,4]. Recent studies have linked ARHL with cognitive decline and dementia, suggesting that the impact of aging on hearing is not limited to peripheral mechanisms but also involves central auditory processing pathways [4,5]. In addition to these neural changes, aging also induces structural changes in the hearing system, such as increased stiffness of the tympanic membrane and ossicular chain, modification of the ear canal shape, reabsorption of ground substances (hyaluronic acid, glucosaminoglycans), decreased function, and changes in adjacent structures, including muscles, cartilage, and tissues [6,7,8].

Among the age-affected structures, the middle ear is particularly important since its vascularity, elasticity, thickness, muscle fibers, and ligament atrophy are all influenced by age [9]. Additionally, aging leads to the decomposition of calcium molecules in the tympanic membrane, ossicular changes, ossification of the ossicles, and reduced elasticity of the ossicular joints in the middle ear [8,10,11]. These changes result in alterations in the middle ear measurements with age. Tympanometry has been an essential tool in diagnosing middle ear pathologies since its introduction [12]. Its strengths lie in assessing middle ear function noninvasively and reliably by measuring the integrity of the tympanic membrane and ossicles based on variations in air pressure [13,14]. Whether tympanometric results are affected by aging remains unclear since several previous studies have found no association between the two [15,16,17,18,19]. However, we hypothesized that age-related structural changes may alter the environment of the middle ear cavity, thereby affecting tympanometric results.

There is also a lack of large-scale studies specifically examining the correlation between age and tympanometric outcomes in the Korean population. This study aimed to fill this gap in the literature by assessing the effects of aging on tympanometric results using data from the Korea National Health and Nutrition Examination Survey (KNHANES).

## 2. Materials and Methods

### 2.1. Study Design

**Figure 1:**
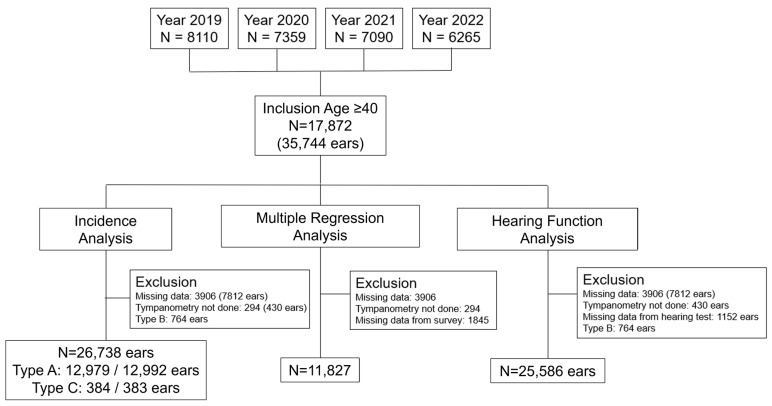
Flowchart of study participant selection, including inclusion and exclusion criteria. Incidence analysis is detailed in Table 1 and Figure 2. Multiple regression analysis is detailed in Table 2. Hearing function analysis is detailed in Figure 3.

A total of 28,824 Korean individuals were screened from the 2019–2022 KNHANES [20]. The inclusion criterion was age ≥ 40 years because tympanometry was included in the health checkup for that age group (Figure 1), and 17,872 individuals with 35,744 ears met the inclusion criteria. The exclusion criteria differed for the three analyses, as shown in Figure 1. All study participants included in the 2019–2022 KNHANES agreed to participate in the survey and signed a consent form. The 2019–2022 KNHANES was approved by the Institutional Review Board of the Korea Centers for Disease Control and Prevention. The approval numbers for the 2019, 2020, 2021, and 2022 surveys were 2018-01-03-C-A, 2018-01-03-2C-A, 2018-01-03-5C-A, and 2018-01-03-4C-A, respectively.

### 2.2. Korea National Health and Nutrition Examination Survey

The KNHANES is a cross-sectional survey, conducted annually in South Korea since 1998. The eighth KNHANES was conducted from 2019 to 2021, and the ninth KNHANES was conducted from 2022 to 2024. However, for the ninth KNHANES, data collection was only completed for the year 2022. The KNHANES sample design was based on a two-stage stratified cluster sampling method (region and household). The participation rates for the eighth and ninth KNHANES were 73.2% and 66.4%, respectively, including 22,559 and 6265 participants.

The KNHANES is divided into three categories: medical survey, nutritional survey, and health examinations. Specifically, we collected datasets from medical surveys and health examinations, which included information on current medical history, smoking status, blood pressure, body mass index (BMI), laboratory test results, and audiological test results. Audiological tests, including impedance audiometry, began in July 2019, resulting in many missing data entries (3906) for that year. To enhance the study’s statistical validity, data from the eighth (2019–2021) and the ninth (2022) KNHANES were merged vertically. The data merging process ensured no duplication by using unique identifiers provided in the survey.

### 2.3. Audiological Test

The participants took a pure-tone audiometry test in an ordinary soundproof booth installed in the vehicle using an AD629 audiometer (Interacoustics, Assens, Denmark). Audiological tests were conducted using an automated pure tone audiometry system employing the Hughson-Westlake procedure [21,22]. In this method, the hearing threshold was determined by requiring participants to respond correctly to 2 out of 3 or 3 out of 5 presentations of a tone. The procedure adjusted the intensity, decreasing by 10 dB after a correct response and increasing by 5 dB after an incorrect response, to accurately record the hearing threshold. Pure-tone audiograms included pure-tone air conduction thresholds, and the average pure-tone threshold (PTA4) was defined as the mean of 500, 1000, 2000, and 4000 Hz frequency thresholds. Tympanometry energy absorbance was measured using Titan IMP440 screeners (Interacoustics, Assens, Denmark) at a 226 Hz frequency. The ear canal air pressure was alternated using a descending pressure sweep from −600 to 300 daPa. The tympanometric results were reviewed by otologists after being initially interpreted by an audiometry tester who is a nurse. A Type A tympanogram was identified if the peak pressure was measured in the range of −100 to 50 daPa, regardless of the peak compliance value. Thus, in this study, both As and Ad types were counted as Type A tympanograms. A Type C tympanogram was identified if the peak pressure was measured below −100 daPa. A Type C tympanogram was identified if the peak pressure was measured below −100 daPa. Cases with ambiguous tympanometric results, which could suggest tympanic membrane perforation or middle ear issues, were classified as missing data.

### 2.4. Medical History and Laboratory Test

We used the results of medical history surveys (otitis media, allergic rhinitis, chronic sinusitis, and obstructive sleep apnea), current smoking status, and laboratory tests (systolic blood pressure, diastolic blood pressure, body mass index, and hemoglobin A1c level). The presence of a physician-diagnosed disease was determined using self-reported medical history surveys. Smoking history was obtained from self-reported surveys; a current smoker was defined as someone who smoked daily at the time of the survey. Blood pressure measurements were performed three times, and the average of the second and third measurements was used in this study. BMI was calculated as body weight (kg) divided by height (m) squared in meters; a BMI of >25 kg/m^2^ indicates obesity. Hemoglobin A1c levels were measured using peripheral blood samples. The laboratory test results were treated as continuous variables.

### 2.5. Statistical Analysis

Linear regression analysis was used to evaluate the statistical significance of the relationship between incidence and age. Multiple logistic regression analysis was performed using the enter method with the Hosmer–Lemeshow test (valid if *p*-value > 0.05) to classify the most associated variables. Nagelkerke’s R^2^ values were calculated to evaluate the explanatory power. Type C tympanogram was included as the dependent variable, and age, sex, current smoking status, allergic rhinitis, chronic sinusitis, obstructive sleep apnea, systolic blood pressure, diastolic blood pressure, body mass index, and hemoglobin A1c level were included as independent variables in the regression model. A two-way analysis of variance (ANOVA) test with Sidak’s multiple comparison test (post hoc) was used to evaluate the statistical significance of the PTA thresholds between the two groups. A *p*-value < 0.05 was considered statistically significant. Statistical analyses were conducted using SPSS version 27.0 (IBM, Armonk, NY, USA).

## 3. Results

**Figure 2:**
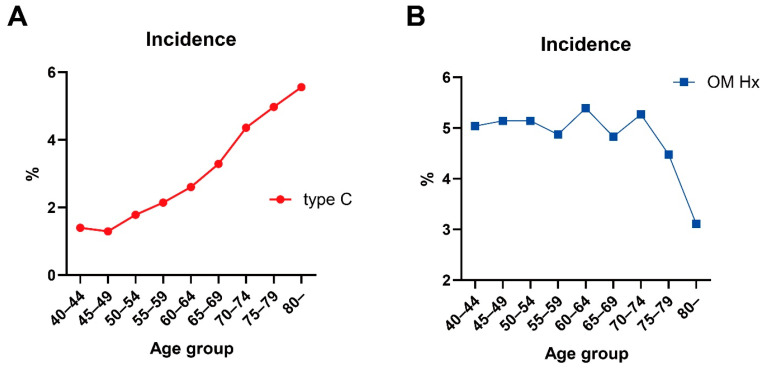
Incidence of type C tympanogram and history of otitis media by age group (**A**) Incidence of Type C tympanograms by age group. (**B**) Incidence of self-reported otitis media history (OM Hx) by age group.

We included 26,738 ears that met the inclusion and exclusion criteria in the study. Based on the tympanometry findings, 25,971 ears (12,979 right ears and 12,992 left ears) had type A, and 767 ears (384 right ears and 383 left ears) had type C tympanogram. Table 1 summarizes the type C tympanogram incidence in the enrolled ears, and Figure 2 shows a histogram of the findings in 5-year age bins. These results showed an increased incidence of type C tympanogram with advancing age, with a correlation coefficient R^2^ of 0.953 (*p* < 0.001). Subsequently, the history of otitis media diagnosis was analyzed to evaluate its relationship with type C tympanogram. However, the history of any kind of otitis media showed a negative correlation with age (R^2^ = 0.393, *p* = 0.071).

**Table 1:** Incidence of type A and C tympanograms according to age.

Age Group (Years)	Type A	Type C	%
40–44	2897	41	1.396
45–49	3138	41	1.290
50–54	3030	55	1.783
55–59	3244	71	2.142
60–64	3592	96	2.603
65–69	3150	107	3.285
70–74	2788	127	4.357
75–79	2160	113	4.971
≥80	1972	116	5.556
Total	25,971	767	2.869

Type A: Number of participants with type A tympanogram in both ears. Type C: Number of participants with type C tympanogram in at least one ear.

Multiple logistic regression analysis was used to evaluate the significance of the correlation between age and type C tympanogram, accounting for other confounding risk factors (Table 2). The included factors were demographics (age and sex), risk of eustachian tube dysfunction (current smoking status, allergic rhinitis history, chronic sinusitis history, and obstructive sleep apnea history), and lifestyle disease markers (systolic blood pressure, diastolic blood pressure, body mass index, and hemoglobin A1c level) [23,24,25,26,27,28,29,30]. Age (*p* < 0.001, odds ratio 1.052) exhibited the most significant correlation with type C tympanogram, whereas other factors were not statistically significant.

**Table 2:** Multiple logistic regression analysis of impedance audiometry type C (N = 11,827, Method = Enter).

Variable	*p*-Value	Odds Ratio	95% CI
Age *	<0.001	1.052	1.042–1.062
Sex ^1^	0.096	1.165	0.973–1.394
Current smoking status ^1^	0.872	0.979	0.753–1.272
Allergic rhinitis ^1^	0.811	0.967	0.731–1.277
Chronic sinusitis ^1^	0.509	0.885	0.617–1.271
OSA ^1^	0.579	0.771	0.308–1.931
SBP	0.570	0.998	0.991–1.005
DBP	0.324	1.006	0.994–1.019
BMI	0.817	0.997	0.971–1.024
HbA1c	0.364	1.048	0.948–1.158

^1^ Categorical variables are defined as follows: sex: male = 1, female = 0; current smoking: no = 1, yes = 0; allergic rhinitis: no = 1, yes = 0; chronic sinusitis: no = 1, yes = 0; OSAS: no = 1, yes = 0. Overall percentage correct = 95.0%. Nagelkerke R square was 0.044. Hosmer–Lemeshow test *p*-value = 0.938. OSA, obstructive sleep apnea; SBP, systolic blood pressure; DBP, diastolic blood pressure; BMI, body mass index; HbA1c, hemoglobin A1c; CI, confidence interval; * *p* < 0.05.

**Figure 3:**
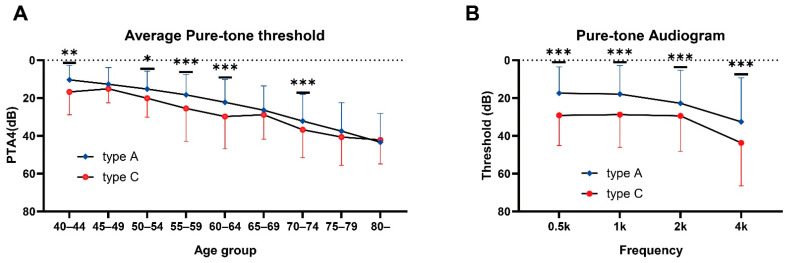
Incidence of type C tympanogram and history of otitis media by age group (**A**) Comparison of average pure-tone threshold (PTA4) between ears with type A and type C tympanograms. (**B**) frequency-specific hearing thresholds in ears with type A and type C tympanograms. Asterisks indicate *p*-values: * < 0.05, ** < 0.01, *** < 0.001.

The average PTA4 was defined as the mean threshold at the frequencies of 0.5, 1, 2, and 4 kHz. The comparison between the PTA4 of ears with type A and type C tympanograms clearly showed worse hearing function in the latter (Figure 3A). In addition, ears with type C tympanogram exhibited significantly poorer hearing than that of ears with type A tympanogram at all frequencies (Figure 3B).

## 4. Discussion

This study aimed to assess the association between age and tympanometric changes, specifically the incidence of type C tympanogram, using data from a large-scale Korean national survey. Our findings revealed a significant correlation between advanced age and the incidence of type C tympanograms.

The increasing incidence of type C tympanograms with age suggests that aging could be a contributing factor in the development of middle ear dysfunction, likely due to age-related physiological changes such as increased stiffness of the tympanic membrane and ossicular chain, decreased acoustic admittance, and reduced eustachian tube function [8,9,31,32]. Specifically, the functioning of the muscles involved in the opening of the eustachian tube, such as the tensor veli palatini (responsible for the rapid opening of the eustachian tube) and levator veli palatini (maintains the eustachian tube opening for longer periods), deteriorates with age. This structural and functional change can lead to negative middle ear pressure, resulting in type C tympanometry findings [33].

In addition to the decreased frequency of eustachian tube opening, age-related changes in the mastoid cavity volume may also play a role in the environmental change of the middle ear. The mastoid bone and its air cells function as air reservoirs and pressure buffers for the middle ear [34,35]. In the Korean population, the mastoid cavity volume peaks in the third decade of life, followed by a gradual decrease in the fourth decade and an abrupt reduction in the seventh decade [36]. This volume reduction may weaken the pressure buffer function of the middle ear, thus contributing to variations in the middle ear pressure and increased prevalence of type C tympanograms.

Moreover, previous studies on the tympanometric findings in different age groups have yielded equivocal results depending on the population size. Studies with larger sample sizes (1240 and 3409 participants, respectively) have identified age-related changes in tympanometric results, consistent with our study [37,38]. These studies reported that tympanometric width tends to increase with age, which can be explained by an increased variation in middle ear pressure. In contrast, studies with smaller sample sizes did not observe these changes [15,18].

Interestingly, our analysis showed a negative correlation between a history of otitis media and age, indicating that tympanometric results did not correlate with a history of otitis media. This finding suggests that the increased incidence of type C tympanograms in older adults is more likely driven by age-related changes in the middle ear and eustachian tube function rather than by past otitis media. This is further supported by a similar correlation observed after controlling for other potential confounders, such as smoking, allergic rhinitis, chronic sinusitis, and lifestyle disease markers.

Based on our findings, we could suggest early detection of type C tympanograms allows for prompt interventions, from medical treatments to improve Eustachian tube function to procedures to equalize middle ear pressure. These interventions can prevent the progression of middle ear dysfunction and subsequent hearing loss. However, this study has some limitations. First, the cross-sectional design of the study prevented us from establishing a clear causal relationship between age and tympanometric results. Second, while tympanometry can serve as an indicator of middle ear pressure, it is not a direct method for measuring pressure. We did not directly measure middle ear pressure, nor did we include bone conduction audiometry or otoscopic findings. The absence of these data limits our ability to fully assess middle ear status and to identify specific middle ear environmental changes that contribute to tympanometric alterations. Future research should explore the mechanisms underlying age-related tympanometric changes and their correlation with eustachian tube dysfunction and middle ear environmental changes. Longitudinal studies incorporating the missing methods mentioned above are needed to establish causality and deepen our understanding of middle ear function in aging populations.

## 5. Conclusions

This large-scale cross-sectional study provides strong evidence that aging is significantly associated with type C tympanograms, indicating increased eustachian tube dysfunction and instability of middle ear pressure. Our findings suggest that tympanometric screening in older adults could be beneficial for and management of middle ear dysfunction, potentially improving hearing outcomes and quality of life in this population. Furthermore, by utilizing extensive big data from the Korean population, our research offers valuable baseline information that can be instrumental for future studies.

## Data Availability

Data supporting the findings of the current study are available from the corresponding author on reasonable request.

## References

[1] Bowl M.R., Dawson S.J. (2019). Age-related hearing loss. Cold Spring Harb. Perspect. Med..

[2] Gates G.A., Mills J.H. (2005). Presbycusis. Lancet.

[3] Jayakody D.M., Friedland P.L., Martins R.N., Sohrabi H.R. (2018). Impact of aging on the auditory system and related cognitive functions: A narrative review. Front. Neurosci..

[4] Peelle J.E., Wingfield A. (2016). The neural consequences of age-related hearing loss. Trends Neurosci..

[5] Griffiths T.D., Lad M., Kumar S., Holmes E., McMurray B., Maguire E.A., Billig A.J., Sedley W. (2020). How can hearing loss cause dementia?. Neuron.

[6] Gaihede M., Koefoed-Nielsen B. (2000). Mechanics of the middle ear system: Age-related changes in viscoelastic properties. Audiol. Neurotol..

[7] Randolph L.J., Schow R.L. (1983). Threshold inaccuracies in an elderly clinical population: Ear canal collapse as a possible cause. J. Speech Lang. Hear. Res..

[8] Ruah C.B., Schachern P.A., Zelterman D., Paparella M.M., Yoon T.H. (1991). Age-related morphologic changes in the human tympanic membrane: A light and electron microscopic study. Arch. Otolaryngol.–Head Neck Surg..

[9] Covell W.P. (1952). Histologic changes in the aging cochlea. J. Gerontol..

[10] Etholm B., Belal A. (1974). Senile changes in the middle ear joints. Ann. Otol. Rhinol. Laryngol..

[11] Harty M. (1953). Elastic tissue in the middle-ear cavity. J. Laryngol. Otol..

[12] Jerger J. (1970). Clinical experience with impedance audiometry. Arch. Otolaryngol..

[13] Holte L. (1996). Aging effects in multifrequency tympanometry. Ear Hear..

[14] Lidén G., Peterson J.L., Björkman G. (1970). Tympanometry. Arch. Otolaryngol..

[15] Chermak G.D., Moore M.K. (1981). Eustachian tube function in the older adult. Ear Hear..

[16] Osterhammel D., Osterhammel P. (1979). Age and sex variations for the normal stapedial reflex thresholds and tympanometric compliance values. Scand. Audiol..

[17] Sinha S.K., Neupane A.K., Gururaj K. (2021). Effect of aging on tympanometric findings in Indian population. Ann. Otol. Neurotol..

[18] Stenklev N.C., Vik O., Laukli E. (2004). The aging ear: An otomicroscopic and tympanometric study. Acta Oto-Laryngol..

[19] Uchida Y., Nomura H., Itoh A., Nakashima T., Ando F., Niino N., Shimokata H. (2000). The effects of age on hearing and middle ear function. J. Epidemiol..

[20] Korea National Health and Nutrition Examination Survey (KNHANES) The Korea National Health and Nutrition Examination Survey (KNHANES), 2019–2022.

[21] Association A.S.-L.-H. (2005). Guidelines for Manual Pure-Tone Threshold Audiometry. https://www.asha.org/policy/gl2005-00014/.

[22] Carhart R., Jerger J.F. (1959). Preferred method for clinical determination of pure-tone thresholds. J. Speech Hear. Disord..

[23] Abd Alhady R., Sharnoubi M.E. (1984). Tympanometric findings in patients with adenoid hyperplasia, chronic sinusitis and tonsillitis. J. Laryngol. Otol..

[24] Ahmed S.T., Lin J., Moskowitz H.S., Stupak H.D. (2021). Can the negative pressures found in obstructive sleep apnea and Eustachian tube dysfunction be related?. Am. J. Otolaryngol..

[25] Dubin M.G., Pollock H.W., Ebert C.S., Berg E., Buenting J.E., Prazma J.P. (2002). Eustachian tube dysfunction after tobacco smoke exposure. Otolaryngol.–Head Neck Surg..

[26] Juszczak H., Aubin-Pouliot A., Sharon J.D., Loftus P.A. (2019). Sinonasal risk factors for eustachian tube dysfunction: Cross-sectional findings from NHANES 2011–2012. Forum Allergy Rhinol..

[27] Lazo-Sáenz J.G., Galván-Aguilera A.A., Martínez-Ordaz V.A., Velasco-Rodríguez V.M., Nieves-Rentería A., Rincón-Castañeda C. (2005). Eustachian tube dysfunction in allergic rhinitis. Otolaryngol.—Head Neck Surg..

[28] Robison J.G., Wilson C., Otteson T.D., Chakravorty S.S., Mehta D.K. (2012). Increased eustachian tube dysfunction in infants with obstructive sleep apnea. Laryngoscope.

[29] Sharma A., MacDowell S., Punjabi N., Kejriwal S., Sharma V., Inman J.C. (2024). Smoking Pack Years and Eustachian Tube Dysfunction. OTO Open.

[30] Stammberger H. (1986). An endoscopic study of tubal function and the diseased ethmoid sinus. Arch. Oto-Rhino-Laryngol..

[31] Blood J., Greenberg H.J. (1977). Acoustic admittance of the ear in the geriatric person. Ear Hear..

[32] Jerger J., Jerger S., Mauldin L. (1972). Studies in impedance audiometry: I. Normal and sensorineural ears. Arch. Otolaryngol..

[33] Tomoda K., Morii S., Yamashita T., Kumazawa T. (1984). Histology of human eustachian tube muscles: Effect of aging. Ann. Otol. Rhinol. Laryngol..

[34] Austin D.F. (1977). On the function of the mastoid. Otolaryngol. Clin. N. Am..

[35] Sade J. (1992). The correlation of middle ear aeration with mastoid pneumatization: The mastoid as a pressure buffer. Eur. Arch. Oto-Rhino-Laryngol..

[36] Lee D.-H., Jun B.-C., Kim D.-G., Jung M.-K., Yeo S.-W. (2005). Volume variation of mastoid pneumatization in different age groups: A study by three-dimensional reconstruction based on computed tomography images. Surg. Radiol. Anat..

[37] McManus B., Harbarger C., Grillis A., Prewitt M.G., Baiduc R., Block D., Paul O., Spankovich C. (2022). Otoscopy and tympanometry outcomes from the National Health and Nutrition Examination Survey (NHANES). Am. J. Otolaryngol..

[38] Wiley T.L., Cruickshanks K., Nondahl D., Tweed T.S., Klein R., Klein B. (1996). Tympanometric measures in older adults. J. Am. Acad. Audiol..

